# Juvenile thyroid malignancy

**DOI:** 10.4103/0971-5851.60054

**Published:** 2009

**Authors:** Sandesh Parelkar, Milind Joshi

**Affiliations:** *Department of Pediatric Surgery, Seth G.S.M.C. and K.E.M. Hospital, Mumbai, India*

**Keywords:** *Juvenile thyroid malignancy*, *papillary carcinoma*, *thyroid nodule*

## Abstract

Thyroid malignancy is an uncommon tumor of the pediatric population. Patients can present with asymptomatic thyroid nodule and it requires thorough work up to rule out the malignancy. Radiological and pathological procedures are a standard part of the management. A 10-year-old girl had asymptomatic thyroid nodule; the cytological examination and the frozen section and final histology of the nodule was different each time. The girl had to undergo total thyroidectomy on the basis of histology of the nodule which was well differentiated papillary carcinoma of thyroid and is under regular follow-up for last two years on thyroid supplementation.

## INTRODUCTION

Differentiated thyroid carcinoma is rare during childhood and adolescence.[1] It constitutes only one to five per cent of pediatric malignancy.[2–5] Nodular presentation is even rarer albeit the malignant potential of the nodule is about 30%.[2] We report the case of a 10-year-old girl who had a thyroid nodule where the findings of fine needle cytology and biopsy were contradictory and it turned out to be papillary carcinoma of thyroid and underwent total thyroidectomy successfully for it.

## CASE REPORT

A 10-year-old girl presented with a single swelling in the front part of neck of six months duration. The swelling was painless and progressive and at the time of examination was 2 × 2 cm on the left side of neck and was moving with deglutition. She had no other complaints and her personal and family history was noncontributory.

**Figure 1:**
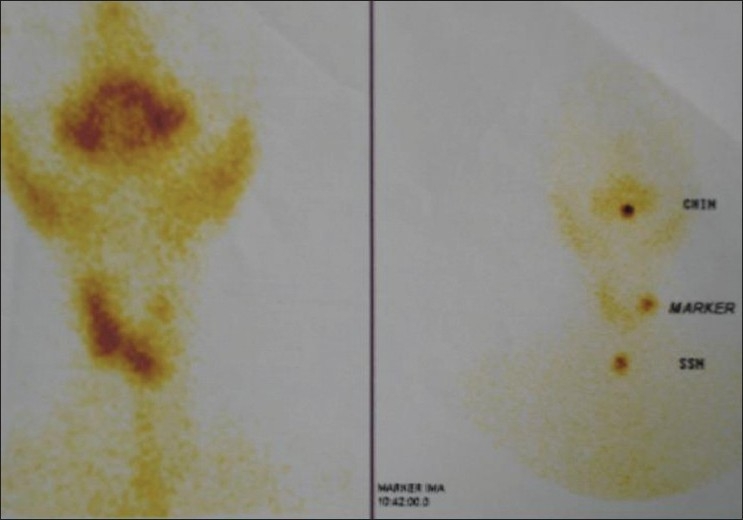
Thyroid nuclear scan showing photopenic area in the region of the thyroid nodule on the left side of the neck

**Figure 2:**
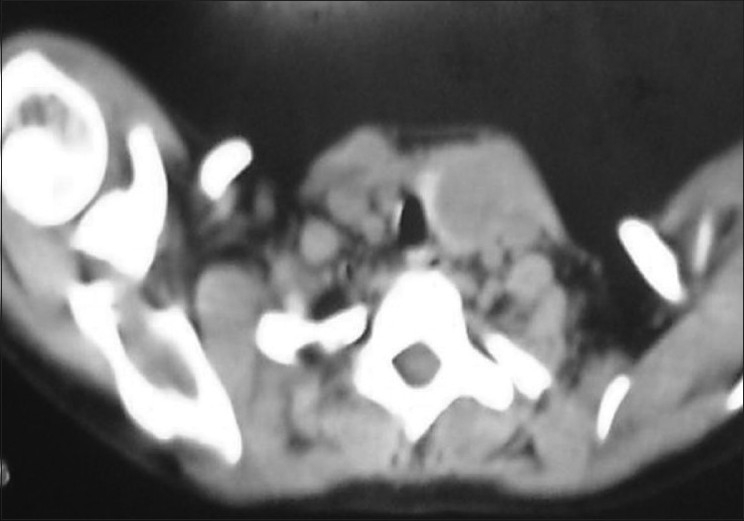
Computerized tomography scan of the neck showing left thyroid nodule

Apart from normal systemic findings and routine investigations, ultrasound of the swelling revealed a mixed solid and cystic swelling of 3 × 2 cm in the left lobe of the thyroid. Nuclear thyroid scan showed cold nodule [Figure 1]. Computerized tomography scan of neck also showed equivocal findings [Figure 2].

**Figure 3:**
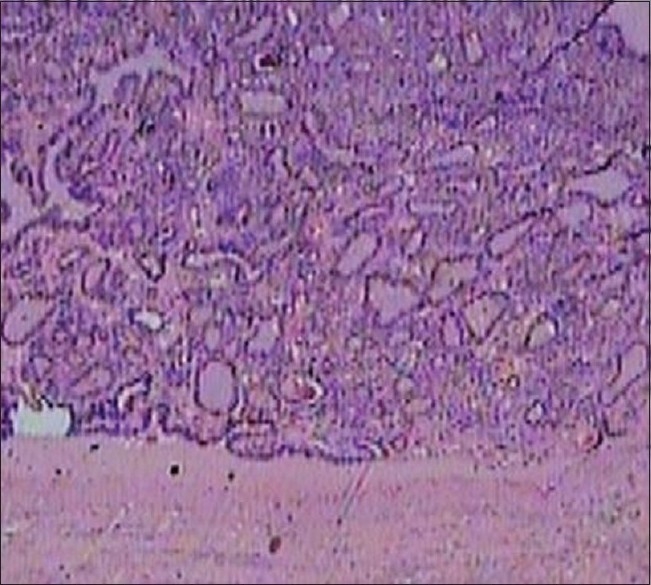
Histopathology of the nodule showing well differentiated thyroid papillary carcinoma, H and E stain, ×10

Fine needle biopsy of the swelling was reported as benign hyper plastic thyroid nodule. Her serum calcitonin level was normal. Left hemithyroidectomy was done. Frozen section examination of the nodule was reported as benign hyper plastic thyroid nodule, surprisingly, however, the final histology report was of a differentiated nodular papillary thyroid carcinoma in the excised thyroid nodule and rest of the lobe and isthmus were normal [Figure 3].

Hence, total thyroidectomy with preservation of the parathyroid was performed after seven days. There was no evidence of any malignant transformation in the rest of the gland on histopathology. Patient was started on thyroid supplementation after her thyroid scan was negative for any residual thyroid tissue after six weeks of surgery. Patient is under regular follow-up since last two years without any symptoms.

## DISCUSSION

Probably no other organ malignancy has greater variation of biologic behavior than pediatric thyroid malignancy.[1,2] Given the relatively well defined histological types of malignancy and proper understanding of the natural history of the specific subtype, one would think about its straight forward management. On the contrary, because of the lack of sufficient comparative studies in the pediatric age group between the type of surgeries for management and relatively slow growth of the common type of thyroid malignancy there is no consensus for its management.[2] Differentiated thyroid carcinomas constitute 95% of the thyroid malignancy of the pediatric age group.[1,2] Medullary thyroid malignancy comprises rest of the five per cent and anaplastic type is very rare.[2,3] Most of the patients are more than 10 years of age and five times female preponderance.[4] Its incidence as second malignancy after the treatment of the first non thyroidal malignancy has decreased because of avoidance of external radiation in children for management of benign or malignant neck conditions.[4] The overall incidence of the primary thyroid malignancy in pediatric age group is 5 cases per million population.[2,5] In most of the previously reported studies, more than fifty percent of the patients PTM and about twenty percent secondary thyroid malignancy had cervical and pulmonary metastasis at the time of initial staging.[5,6]

The role of RET proto oncogene mutation has been well proven in the pathogenesis of this malignancy and with multiple endocrine neoplasia syndrome (MEN).[2] When the clinical presentation is that of a thyroid nodule ,it requires careful evaluation because of about 30% incidence of malignant potential.[2] The laboratory evaluation and thyroid scinti scanning have limited usefulness for the accurate distinction between benign and malignant lesions. Thirty per cent of the cold nodules in the thyroid scan have malignant lesion. Ultrasonography provides excellent distinction between solid and cystic lesion and presence of the solid nodule should prompt the further work up to rule out malignancy. However, presence of cystic lesion is not full proof of benign condition as they can also harbor carcinoma.[7]

Computerized tomography (CT) scan of the neck is useful to detect lesion less than 1 cm, to know the status of the nodes, to differentiate simple or complex nodular lesion. It also cannot define malignant or benign condition very reliably.[2]

Needle biopsy of the thyroid nodule has been recommended for the diagnostic purpose. However, the problem of not getting the proper tissue sample and high incidence of false results limits its finality.[8] Best diagnosis is established by surgical resection and histological examination.[9] In recent years, children with small single lesions of 2 cm or less underwent lobectomy and isthmectomy. Total thyroidectomy or near total thyroidectomy with or without lymph node dissection is done when the tumour was large or when there was marked cellular dysplasia or tumour invasion beyond the thyroid capsule. Modified radical neck dissection is done when the nodal involvement is confirmed by histology.[9,10] In patients, after total thyroidectomy, serum thyroglobulin can be used as a tumor marker for diagnosing the recurrence of the disease.[2–4] As described by Candy and Crile, low risk thyroid nodule defined as less than 2cm in size, showing presence of well differentiated tumour histology and no other synchronous lesion are easily managed by lobectomy and isthmectomy and proper follow up.[5,6]

For larger lesion and follicular and medullary carcinoma, syndromic association (MEN) and capsular involvement and positive neck nodes total thyroidectomy with neck dissection is necessary followed by radioactive iodine therapy.[3–6] In our patient, the diagnostic inaccuracy and contradictory results of the needle biopsy, frozen section, CT scan of the thyroid and thyroid nuclear scan findings limited our initial options for the management of this patient but we always had the doubt of malignancy and confirmatory histology lead us to total thyroidectomy and thyroid supplementation postoperatively with satisfactory result.
